# Organisational Culture Predictors of Job Satisfaction Among Nurses in Kenya, Sub‐Sahara Africa: A Cross‐Sectional Study

**DOI:** 10.1002/nop2.70298

**Published:** 2025-09-10

**Authors:** Evans Kasmai Kiptulon, Anna Szőllősi, Zrínyi Miklós, Adrienn Ujváriné Siket

**Affiliations:** ^1^ Doctoral School of Health Sciences, Faculty of Health Sciences University of Pécs Pécs Hungary; ^2^ The Kenya Medical Training College Nairobi Kenya; ^3^ Doctoral School of Health Sciences, Faculty of Health Sciences University of Debrecen Debrecen Hungary

**Keywords:** job satisfaction, Kenya, nurses, organisational culture

## Abstract

**Aims:**

To find out how Kenyan nurses rate their organisational culture, determine their level of job satisfaction, and organisational culture predictors of job satisfaction.

**Design:**

A cross‐sectional online survey.

**Methodology:**

A total of 300 nurses across Kenya were invited to participate in this study. The Practice Environment Scale of Nursing Work Index was used to measure organisational culture, while a single‐item scale was used to assess job satisfaction. Data analysis was done using SPSS version 28. Chi‐square, correlation, and bivariate logistic regression were performed to determine the relationship among the study variables.

**Results:**

Kenya's overall organisational culture was rated as weak‐positive (*M* = 2.51). Only 24.5% of nurses reported being satisfied, while 74.5% were dissatisfied or neutral. Structural Equation Modelling of the five sub‐scales demonstrated an acceptable fit and accounted for 43.7% of job satisfaction variance. Significant predictors of job satisfaction included participation in hospital affairs (β = 0.409, SE = 0.084, *T* = 5.396, *p* < 0.001) and staffing and resource adequacy (β = 0.311, SE = 0.060, *T* = 4.665, *p* < 0.001).

**Conclusion:**

This study highlights the crucial role of organisational culture in determining nurses' job satisfaction. It provides robust evidence to policymakers, hospital administrators, nurse managers and other health stakeholders of the need to fully involve and engage nurses in hospital affairs while providing adequate staffing and resources. Such measures are essential for creating a highly satisfied workforce, fostering the delivery of high‐quality nursing care to patients.

Nurse Managers and hospital management should strive to continuously monitor and build positive organisational cultures to raise the quality of service delivery and retain the nursing workforce.

**Patient or Public Contribution:**

This study involves no patient or public contribution.

**Abbreviations**
BScN: Bachelor of Science in Nursing
FQC: foundations for quality of care
HRH: human resource for health
JS: job satisfaction
LS: leadership and support
MoH: Ministry of Health
NPR: nurses physician relations
OC: organisational culture
PES‐NWI: Practice Environment Scale of Nursing Work Index
PHA: participation in hospital affairs
PhD: Doctor of Philosophy
SD: standard deviation
SEM: structural equation modelling
SPSS: statistical package for the social sciences
SRA: staffing and resources adequacy

## 1 Introduction

Nursing is the largest healthcare profession, with nearly 27.9 million personnel, accounting for 59% of the total health workforce worldwide (Buchan 2023). In Kenya, there were 81,564 nurses in 2022 (Statista 2022), which represents more than 50% of the total health workforce in the country. Therefore, paying attention to and prioritising job satisfaction among nurses is of paramount importance, as it will guarantee better health for the entire nation. According to studies conducted in Kenya (Chepkemoi 2020; Nyang'echi et al. 2019; Sang et al. 2021; Aruasa et al. 2019), nursing is one of the largely dissatisfied healthcare professions within the healthcare sector, consistently showing low levels of job satisfaction. This is further evidenced by high nurse turnover (Afulani et al. 2021; Kirigia et al. 2006; Sang et al. 2021), protests, strikes (Gathongo and Ndimurwimo 2020; Kaguthi et al. 2020) and a series of industrial actions done by nurses in the country in pursuit of government action in solving problems bedevilling them.

Job satisfaction is a complex and multi‐faceted concept. To date, there is still no general agreement among researchers and scholars on the precise definition of what job satisfaction is. The first definition of job satisfaction was provided in1935 by Robert Hoppock, who described it as ‘any combination of psychological, physiological, and environmental circumstances that cause a person to truthfully say, I am satisfied with my job’(Brikend Aziri 2011; Hoppock and Spiegler 1935). This foundational definition has paved the way for numerous other definitions and perspectives. For example, Boumans and Landeweerd (1994) described it as ‘the overall measure of the degree to which employees are satisfied and happy with their job’(Boumans and Landeweerd 1994), liking or enjoying one's job (McDonald et al. 2012), positivity about the work experience (Sexton et al. 2006), the feeling of contentment formed by how a job is perceived by an individual and is one of the most significant requirements for success and productivity (Lin et al. 2012), a pleasurable or positive emotional state resulting from the appraisal of one's job or job experiences (Judge 2017), the extent to which an employee is happy with his/her job (Oshagbemi 1999) among many other definitions. For purposes of this study, we defined job satisfaction as ‘how nurses in Kenya are happy, like, enjoy, feel positive or are contented with their nursing job’.

The critical importance of job satisfaction in nursing and healthcare has made it one of the most heavily studied concepts worldwide. Job satisfaction is a key indicator of well‐being for nurse employees (Wang et al. 2012). It improves nurses' mental, physical, and emotional health, thereby reducing work‐related stress and burnout, which are highly prevalent problems in nursing associated with job dissatisfaction (Hayes et al. 2009; Judge 2017; Widodo et al. 2021; Zhang et al. 2014). Researchers have argued that burnout and work‐related stress caused by job discontentment, among other factors, have a detrimental consequence on nurses. These effects range from mild, like insomnia, anxiety, and emotional exhaustion, to major effects like panic attacks, depression, and mental disorders (Kaushik et al. 2021; Nagel and Nilsson 2022). In addition, they weaken nurses' immunity, predisposing them to gastrointestinal diseases (stomach ulcers, heartburns, and indigestion), several cardiovascular diseases (Alenezi et al. 2018), musculoskeletal disorders, back pain, headache, obesity, and other chronic diseases including hypertension (Aghaei and Asadi 2020). Job satisfaction also enhances nurses' work productivity, performance efficiency, effectiveness, and health service quality improvement, which is a precursor for superior patient outcomes (Chepkemoi 2020; Dirdjo et al. 2023). Apart from improving nurses' mental health and well‐being, job satisfaction also enhances nurse retention by lowering career and professional turnover and turnover intentions (Alharbi et al. 2020; Zahednezhad et al. 2021; Zhang et al. 2014). It increases nurses' organisational and career commitment (Gary et al. 2023; Hakami et al. 2020). This significantly lowers job absenteeism. Furthermore, it fosters a positive work culture like teamwork, unity, communication and collaboration (Bragadóttir et al. 2023).

Across global healthcare systems, job satisfaction has been shown to be predicted by organisational culture (Akinwale and George 2020; Aloisio et al. 2021; Al‐Surimi et al. 2022; Bellou 2010; Choi et al. 2014; Choi et al. 2013; Hayes et al. 2009; Hulkova et al. 2024; Hwang 2019; Labrague et al. 2017; Labrague et al. 2017; Park and Kim 2009; Roza et al. 2022; Tsai 2011; Widodo et al. 2021; Zhang et al. 2014). Organisational culture (OC) is defined as ‘a system of shared values, beliefs and morals that produces norms of behaviour and establish an organisational way of life’(Koberg and Chusmir 1987). Rob Goffee and Gareth Jones in 1998 further alluded that OC is ‘simply how we do things around here’(Goffee and Jones 1998). OC is a management concept that centres around leadership (Schein 2018). It is believed that (OC) and leadership are the two sides of the same coin (Chong et al. 2018). It acts as a fabric and glue that binds workers together (Körner et al. 2015). Schein exclaimed that organisational culture consists of artefacts, beliefs, assumptions, and shared values and morals learned by employees as they work, and over time, they become unconsciously part of them (Schein 2018). Researchers have further refined definition and classification of OC in nursing as either positive (magnet) or negative (non‐magnet) (Buffington et al. 2012; Haller et al. 2018; Lake 2002; Widodo et al. 2021). A positive (magnet culture) is one in which nursing is centred on quality of care, cohesive, supportive leadership, adequate resources, and high collegial nurse‐physician relationships, including collaboration, teamwork, inter‐professional collaboration, flexibility of work‐schedules, respect and recognition of nurses, fair reward systems, adequate renumeration, nurses' involvement in hospital affairs, being listened to, continued medical education, and consultation on day‐to‐day activities among others (Banaszak‐Holl et al. 2015; Haller et al. 2018; Queen et al. 2022; Widodo et al. 2021). A negative OC lack these components of positive OC.

Organisational culture has been receiving increasing attention in the recent past over its influence on job satisfaction among nurses. Researchers, for example, Widodo et al. (2021) examining the effects of organisational culture types (orientation culture, consistency culture, involvement culture, and adaptability culture) found a positive effect of organisational culture (c.r. 5.048, *p* = 0.001) and nurse pay satisfaction (c.r. 3.713, *p* = 0.001) on nurse job satisfaction (Widodo et al. 2021). A Korean study by Park and Kim 2009 investigating the same concept of whether different types of organisational culture (consensual culture, developmental culture, hierarchical culture and rational culture) are associated with job satisfaction and turnover intention found a direct association. Among the different types of culture, *consensual culture* (characterised by collaboration, mutual agreement, and shared decision‐making) and *rational culture* (emphasises efficiency, evidence‐based practices, and structured decision‐making processes) had significant, positive associations with the nurses' job satisfaction (Park and Kim 2009). Similarly, Choi et al. (2022) studying the effects of manager competency (staff advocacy and development, team communication, collaboration, change and resource management, quality monitoring, pursuance and personal mastery) which are elements of OC, demonstrated that manager competency was a significant predictor of staff nurses' job satisfaction and turnover intention. This study was closely reflected by Galletta et al. (2011) who argued that supervisor and organisational support act differently as moderators in a relationship between care adequacy–job satisfaction–turnover intention. Job satisfaction also in this study was a key mediating variable between care adequacy and turnover intention among nurses. A study done in Australia (Hayes et al. 2009) suggested that a positive organisational work environment, characterised by nurses feeling valued, flexible management support, and availability of opportunities for career and professional development had a positive correlation with job satisfaction and personal accomplishment, while it had negative correlations with job stress, emotional exhaustion, and depersonalisation. Aghaei and Asadi (2020), investigating the influence of organisational culture on nurse resilience to occupational stress, job satisfaction, and burnout found a significant positive correlation between organisational culture and job satisfaction (*r* = 0.29) and resilience (*r* = 0.21). There was a significant negative correlation between positive organisational culture and occupational stress (*r* = −0.22), and burnout (*r* = −0.14). A group of other studies have also found that aspects of organisational culture and environmental components like professionalism, relationships with co‐workers, management, staffing and resources adequacy, and ward practices (Choi et al. 2014, 2022), organisational cultural values, including fairness, opportunities for growth, and the organisation's reputation (Sharma 2017), relationships at the workplace, communication in the team or with a superior (Hulkova et al. 2024) largely affect job satisfaction among nurses. Several studies from Thailand (Nantsupawat et al. 2017), South Africa (Ditlopo et al. 2024), Turkey (Arslan Yurumezoglu and Kocaman 2016), Australia (Hayes et al. 2009), Finland (Eskola et al. 2016), examining organisational work environment parameters such as nurse participation in hospital affairs, nursing foundations for quality of care, manager ability, leadership, and support of nurses, staffing and resource adequacy and collegial nurse‐physician relations, found that high and strongly positive scores, measured using the Practice Environment Scale of Nursing Work Index, were consistently associated with higher levels of job satisfaction, greater retention of nurses at workplace, and improved overall morale among nurses. Conversely, low and poor ratings of these variables of OC were strongly associated with job dissatisfaction, increased turnover intentions, and diminished job morale. Similarly, studies from Sub‐Sahara Africa; Nigeria (Olusegun Emmanuel and George 2020) and Ethiopia (Ayalew et al. 2019) have also shown similar results with these studies.

The problem of nurses' job satisfaction in Kenya remains a fundamental issue that has prevented quality service delivery, particularly in both national and county hospitals in the country. Many researchers have shown that job satisfaction among nurses is critically low (Chepkemoi 2020; Gathongo and Ndimurwimo 2020; Nyang'echi et al. 2019; Sang et al. 2021). This has been associated with Kenya experiencing a significant ‘brain drain’ with highly skilled nurses exiting the country en masse in search of better opportunities and healthier working conditions abroad. Initially, monetary incentives including good wages and compensation were considered the only way to promote job satisfaction. Many studies done in Kenya have focused on this and other variables in relation to job satisfaction. Despite the critical role that organisational culture plays in nurses' job satisfaction, there is a paucity of dedicated studies conducted in the country. Therefore, this research aimed to fill this gap.

### 1.1 The Aim of This Study

To find out how Kenyan nurses rate their organisational culture.

To determine the level of job satisfaction among nurses in Kenya.

To find out the organisational culture predictors of job satisfaction among nurses in Kenya.

The study was guided by the following null hypotheses:

Hypothesis 1 *There is no significant relationship between nurse participation in hospital affairs and nurses' job satisfaction*.

Hypothesis 2 *There is no significant relationship between nursing foundations for quality of care and nurses' job satisfaction*.

Hypothesis 3 *There is no significant relationship between nurse manager's ability, leadership, and support of nursing and nurses' job satisfaction*.

Hypothesis 4 *There is no significant relationship between staffing and resource adequacy and nurses' job satisfaction*.

Hypothesis 5 *There is no significant relationship between collegial nurse‐physician relations and nurses' job satisfaction*.

## 2 Methodology

### 2.1 Study Design, Location and Population

This was a cross‐sectional online survey done among 300 hospital nurses working in various health facilities in Kenya. Kenya is a middle‐income country in Sub‐Saharan Africa located on the continent's eastern coast. Health services are provided by a comprehensive network of healthcare facilities—more than 9696 across the country, according to the Kenya Ministry of Health's database (MOH KENYA 2020). There are various levels of these healthcare facilities: ranked from the lowest level to the highest, these facilities are namely community level, dispensaries, health centres, sub‐county hospitals, county hospitals, and national referral hospitals. Nurses working in these facilities hold different qualifications, with a certificate in nursing being the lowest nursing qualification and a PhD being the highest qualification. These qualifications chronologically are certificate in nursing, diploma in nursing, bachelor's degree in nursing (BScN), masters, and PhDs. According to the Nursing Council of Kenya and the Ministry of Health Statistics 2023, the country had a total of 81,564 nurses in three major compositions of 39,458 certificate/enrolled nurses, 32,169 diploma nurses, and 9937‐degree (BScN) nurses (Statista 2022) number of nurses has been progressively increasing in the country.

### 2.2 The Sample Size Determination

The desired sample size for this study was calculated using the ‘rule of thumb formula’ as used by other studies (AbuAlRub and Nasrallah 2017; Wilson Van Voorhis and Morgan 2007). The rule states that at least 200 sample size or having 5–10 participants per parameter is an adequate sample size for research in structural equation modelling. Our main data collection tool (PES‐NWI) had 31 items; thus, the adequate sample size should be between 31 × 5 = 155 (minimum) and 31 × 10 = 300 (the maximum). Therefore, we sent the online questionnaire through email to 300 nurses working in Kenya's county and national referral hospitals, and major private hospitals, inviting them to participate in the research.

### 2.3 Sampling Procedure

A multi‐stage sampling technique was used. First, there was the selection of the participating regions and hospitals. To perform this process, Kenya was subdivided into 8 regions as per the former provincial administrative units, namely Nairobi, Coast, Eastern, Rift Valley, Northeastern, Central, Nyanza, and Western provinces. Using simple random sampling by a computer number generator, four provinces—Nairobi, Central, Western, and Nyanza were—selected. A list comprising major private hospitals, county and national referral hospitals within the selected regions was generated, giving a total of 36 major hospitals in the first phase. However, 21 hospitals agreed to participate in the data collection. A convenience sampling method was used to recruit the nurses who participated in this study. A link to an online survey questionnaire and a request to participate in the research was shared with the nurses through email or individual WhatsApp.

### 2.4 Inclusion and Exclusion Criteria

All nurses irrespective of their cadre, licenced by the Nursing Council of Kenya, who have worked in the selected hospitals for at least twelve uninterrupted months and consented to the study were included. Those who did not consent, part‐time nurses, nurses with pending disciplinary action and nurse interns were excluded from this study.

### 2.5 Data Collection Process, Instruments and Measurements

*Organisational culture*: The Practice Environment Scale of Nursing Work Index (PES‐NWI) (31‐item scale) (Lake 2002) questionnaire was used to measure organisational culture. The PES‐NWI‐31 measures hospital work environment culture in 5 main sub‐scales: *Nurse participation in hospital affairs (items 1–9), Nursing foundations for quality of care (items 10–19), Manager ability, leadership, and support of nurses (items 20–24), Staffing and adequacy of resources (items 25–28)* and *Collegial nurse‐physician relations (items 29–31)*. These questions are answered on a 4‐point Likert scale: 1 = strongly disagree, 2 = disagree, 3 = agree, 4 = strongly agree. To analyse the PES‐NWI tool, an average score is computed for each single item sub‐scale and the overall/composite scale. Higher scores above the midpoint of 2.5 represent a positive/magnet nursing practice environment culture (Lake 2002), and a lower score below the midpoint of 2.5 characterises a negative nursing organisational culture. A magnet culture is one in which nursing is centred on quality of care, cohesive, supportive leadership, adequate resources, and high collegial nurse‐physician relationships, including collaboration, teamwork, and inter‐professional collaboration. Other characteristics of this culture include nurses' involvement in hospital affairs, being listened to, continued medical education, and consultation on day‐to‐day activities. A negative organisational culture is the opposite of positive OC.

*Job satisfaction*: Job satisfaction was measured with a single item ‘Describe your overall satisfaction with nursing organisational culture and work environment’. Use of a single‐item measure to assess overall job satisfaction has been well supported in literature with several studies demonstrating that a single‐item measure is both valid and reliable to assess job satisfaction (Dolbier et al. 2005; Fakunmoju 2021; Wanous et al. 1997). The job satisfaction question answers were given on a 5‐Likert scale (1 = very dissatisfied, 2 = dissatisfied, 3 = neutral,4 = satisfied, 5 = very satisfied). During the data collection process, the nurses were first asked to fill in the PES‐NWI‐31 and thereafter, the job satisfaction question was asked. This was designed to get an overall perception of job satisfaction in regard to the 5 sub‐scales of PES‐NWI‐31.

### 2.6 Data Analysis

The data was downloaded from an online Google form Excel and then imported into the Statistical Package for the Social Sciences (SPSS) version 28 for analysis. Descriptive statistics, including frequencies, percentages, means, and standard deviations, were used to assess the demographic parameters. Correlation and linear regression through Structural Equation Modelling (SEM) were used to test the hypothesis and find out the organisational culture determinants of job satisfaction.

## 3 Results

### 3.1 Participant's Demographic Characteristics

Out of the 300 invited nurses, 212 participated in this study, resulting in a 70.7% response rate. Table 1 presents the demographic characteristics of the participants, including gender, age, marital status, years of work experience, religion, highest education level, employment type and designation. The mean age of the participants was 30 years (SD = 8.53), with the youngest participant being 22 years old and the oldest 59. The sample consisted of 57.5% female and 42.5% male. The mean work experience was 10.33 years, with the majority (*n* = 127) employed on permanent and pensionable terms. Regarding the highest education level, a significant number of the nurses held either a diploma (47.6%, *n* = 101) or a degree (30.7%, *n* = 65). Additionally, some participants held certificates in nursing (*n* = 15), and master's degrees (*n* = 30), and one respondent had a PhD. More than 97% were Christians.

**TABLE 1:** Participant's demographic characteristics (*n* = 212).

Variables		Frequency (*n*)	(%)
Gender	Male	90	42.5
	Female	122	57.5
Marital Status	Married	152	71.7
	Single	57	26.9
	Widow/Widower	3	1.4
Age	21–30	76	35.8
	31–40	95	44.8
	41–50	28	13.2
	51–60	13	6.1
Years of Experience	≤ 5 years	71	33.5
	6–15 years	102	48.1
	16–25 years	25	11.8
	≥ 26 years	14	6.6
Religion	Christian	207	97.6
	Muslim	4	1.9
	Others	1	0.5
Highest education	Certificate in Nursing	15	7.1
	Diploma in Nursing	101	47.6
	BScN	65	30.7
	Masters	30	14.2
	PhD	1	0.5
Type of employment	Permanent & pensionable	127	59.9
	Fixed term Contract	64	30.2
	Part‐time job	21	9.9
Designations	Nurse supervisor/manager	45	21.2
	The nurse ward head/Nursing in‐charge	38	17.9
	General nursing Staff	128	60.4

### 3.2 Nurses' Perception of Organisational Culture in Kenyan Healthcare Facilities

Using PES‐NWI (Lake 2002), of average mean scores above 2.5 to mean positive OC and under 2.5 to mean negative OC, the nurses rated their organisational culture and environment characteristics. The scores presented in Table 2 show individual item ratings, sub‐scale, and the overall rating of the organisational culture in Kenya. Overall, the nurses rated the organisational culture as moderately positive, although it was at the lowest end of the positive range (*M* = 2.51). The sub‐scale of *staff and resource adequacy* had the lowest mean score (*M* = 2.11, SD = 1.06), with all items scoring below 2.5. This is interpreted to mean there is an acute lack of staff, including nurses, to get work done in Kenyan hospitals. Furthermore, the nurses are overwhelmed with work and rarely get time to discuss patient issues with colleagues (Item 28, *M* = 2.39). *Nurse participation in hospital affairs* sub‐scale had the second lowest mean score (*M* = 2.45, SD = 1.03). Of the nine items in this sub‐scale, 7 were scored below 2.5, a major communication that nurses are not fully involved in hospital affairs. Deeper individual item analysis shows that involvement in governance (*M* = 2.39) and policymaking (2.37) were scored poorly. The result furthermore in this sub‐scale indicates that there is a lack of professional development and other career opportunities, as shown by opportunities for training and academic progress (*M* = 2.36), career promotions and ladder rise (*M* = 2.22). The nurses also indicated that nurse managers and executives have unequal power and authority compared to other professional executives within the same hospital. On average mean, the other sub‐scales scored positive, with *nursing foundations for quality‐of‐care scoring* (*M* = 2.64, SD = 1.01) *nurse manager ability, leadership, and support of nurses* (*M* = 2.63, SD = 1.00) and *collegial nurse‐physician relations* (*M* = 2.67, SD = 0.92). On individual item analysis within these 3 sub‐scales, only collegial nurse‐physician relations had all items score highly positive. This shows the strength of collaboration, teamwork, and good working relationships between nurses, doctors, and other professionals. Even though 16 items out of 31 were scored below 2.5, some items were scored strongly positive, including high standards of nursing care expected by the administration (*M* = 3.16), working with clinically competent nurses (*M* = 3.27) and head nurse is a good manager (*M* = 3.07).

**TABLE 2:** Rating of organisational culture of the work environment *n* (%) *n* = 212.

Sn	Item	Strongly disagree	Disagree	Agree	Strongly agree	Mean (SD)‐4 Scales
Sub‐Scale 1: Nurse participation in hospital affairs 2.45 (1.03)						
1	Staff nurses are involved in the internal governance of this hospital	57 (26.9)	52 (24.5)	67 (31.6)	36 (17)	2.39 (1.06)
2	Staff nurses participate in policy decisions that affect day‐to‐day work in this hospital	58 (27.4)	58 (27.4)	56 (26.4)	40 (18.9)	2.37 (1.08)
3	In this hospital, there are many opportunities for advancement of nursing personnel through professional training and other academic opportunities.	57 (26.9)	63 (29.7)	50 (23.6)	42 (19.8)	2.36 (1.08)
4	In this hospital, we have an administration which listens to and responds to employees' concerns.	54 (25.5)	63 (29.7)	60 (28.3)	35 (16.5)	2.36 (1.04)
5	The Head of the nursing department (Nursing director/manager) of this hospital is highly visible and accessible.	17 (8.0)	43 (20.3)	83 (39.2)	69 (32.5)	2.96 (0.92)
6	In this hospital, there is opportunity for Career development/clinical ladder rise for nurses through for example, through promotions	67 (31.6)	61 (28.8)	54 (25.5)	30 (14.2)	2.22 (1.05)
7	Nurse administrators/managers consult with nurses over problems arising in the day to day working routine procedures.	39 (18.4)	49 (23.1)	78 (36.8)	46 (21.7)	2.62 (1.02)
8	In this hospital, Staff nurses have the opportunity to serve on hospital and nursing department committees	42 (19.8)	60 (28.3)	66 (31.1)	44 (20.8)	2.53 (1.03)
9	Chief nursing executive or Nurse managers in this hospital have equal power and authority like the other top level hospital executive.	67 (31.6)	65 (30.7)	51 (24.1)	29 (13.7)	2.20 (1.03)
Sub‐Scale 2: Nursing foundations for quality of care						2.64 (1.01)
10	In this hospital, Nursing diagnoses is seriously considered important in the management of patients	40 (18.9)	52 (24.5)	70 (33)	50 (23.6)	2.61 (1.05)
11	In this hospital, there is an active quality assurance programme to ensure high‐quality nursing care	38 (17.9)	54 (25.5)	76 (35.8)	44 (20.8)	2.59 (1.01)
12	In this hospital, there is a preceptor programme for newcomers to welcome, brief and give them the support needed to settle for example, orientation and some training.	41 (19.3)	50 (23.6)	69 (32.5)	52 (24.5)	2.62 (1.06)
13	Nursing care in this hospital is based on a nursing model, rather than a medical, model.	53 (25)	48 (22.6)	71 (33.5)	40 (18.9)	2.46 (1.06)
14	In this hospital, Patient care assignment fosters or promotes continuity of care (e.g., the same nurse cares for the patient from one day to the next).	59 (27.8)	51 (24.1)	65 (30.7)	37 (17.5)	2.38 (1.07)
15	In this hospital, there exists a common, well‐defined nursing philosophy in place that pervades the patient care environment that is felt and is spread across the patient care environment	43 (20.3)	51 (24.1)	81 (38.2)	37 (17.5)	2.53 (1.00)
16	In this hospital, written, up‐to‐date nursing care plans for all patients are always done.	72 (34.0)	57 (26.9)	49 (23.1)	34 (16)	2.21 (1.08)
17	High standards of nursing care are expected by the administration or managers at this hospital.	13 (6.1)	37 (17.5)	66 (31.1)	96 (45.3)	3.16 (0.92)
18	In this hospital, there is an active in‐service/continuing education programme for nurses.	48 (22.6)	43 (20.3)	84 (39.6)	37 (17.5)	2.52 (1.03)
19	In this hospital I am working with nurses who are clinically competent.	12 (5.7)	16 (7.5)	86 (40.6)	98 (46.2)	3.27 (0.83)
Sub‐Scale 3: Nurse manager ability, leadership, and support of nursing						2.63 (1.00)
20	In this hospital or the ward unit where I work, the Head Nurse is a good manager and leader.	20 (9.4)	25 (11.8)	87 (41)	80 (37.8)	3.07 (0.93)
21	The head nurse/supervisor in this hospital backs up the nursing staff in decision‐making, even if the conflict is with a physician.	34 (16.0)	48 (22.6)	78 (36.8)	52 (24.5)	2.70 (1.01)
22	Head nurses or Supervisors use mistakes and errors made by juniors as learning opportunities, not criticism.	48 (22.6)	54 (25.5)	70 (33.0)	40 (18.9)	2.48 (1.04)
23	In this hospital, we have supervisors/supervisory team that is supportive to nurses that is, supportive supervision and ready to help	36 (17.0)	56 (26.4)	81 (38.2)	39 (18.4)	2.58 (0.98)
24	In this hospital, the management gives praise and recognition for a job well done by nurses.	56 (26.4)	63 (29.7)	63 (29.7)	30 (14.2)	2.32 (1.02)
Sub‐Scale 4: Staffing and Resource Adequacy 2.11 (1.06)						
25	In this hospital, there are enough staff and personnel to get the work done.	108 (50.9)	52 (24.5)	33 (15.6)	19 (9.0)	1.83 (1.00)
26	In this hospital, there are enough number of qualified nurses to provide quality patient care.	94 (44.3)	47 (22.2)	43 (20.3)	28 (13.2)	2.02 (1.09)
27	In this hospital, there are adequate support services to allow me to spend time with my patients (Services e.g., hospital support staff, administrative staff, etc. that prevent you from doing non‐nursing activities.)	72 (34.0)	57 (26.9)	53 (25.0)	30 (14.2)	2.19 (1.06)
28	In this hospital, I get enough time and opportunity to discuss patient care problems with other nurses.	55 (25.9)	58 (27.4)	60 (28.3)	39 (18.4)	2.39 (1.08)
Sub‐Scale 5: Collegial nurse–physician relations 2.67 (0.92)						
29	In this hospital, there is a lot of teamwork between nurses and doctors.	26 (12.3)	57 (26.9)	87 (41.0)	42 (19.8)	2.68 (0.93)
30	In this hospital, physicians and nurses have good working relationships.	25 (11.8)	55 (25.9)	91 (42.9)	41 (19.3)	2.70 (0.91)
31	In this hospital, there is a Functional collaboration (joint practice) between nurses and physicians.	29 (13.7)	57 (26.9)	88 (41.5)	38 (17.9)	2.64 (0.93)
Overall rating of the organisational culture						2.51 (1.01)

### 3.3 Job Satisfaction Among Nurses in Kenya

From the single item ‘Describe your overall satisfaction with nursing organisational culture and work environment aspects’ that was used to elicit job satisfaction among the participants, the results were as shown in Table 3.

**TABLE 3:** How satisfied are you with your nursing organisational work environment?

Satisfaction level	Frequency (*n*)	Percentage
Very dissatisfied	34	16.0
Dissatisfied	46	21.7
Neutral	80	37.7
Satisfied	38	17.9
Very satisfied	14	6.6

Job satisfaction among Kenyan nurses was further re‐classification into 3 major levels; dissatisfied (formed by those who responded ‘very dissatisfied + dissatisfied’ *n* = 80, 37.7%), neutral (remained unchanged *n* = 80, 37.7%) and satisfied (formed by those who responded satisfied + very satisfied *n* = 52, 24.5%). This classification indicated that only 24.5% (*n* = 52) of Kenyan nurses were satisfied with their job.
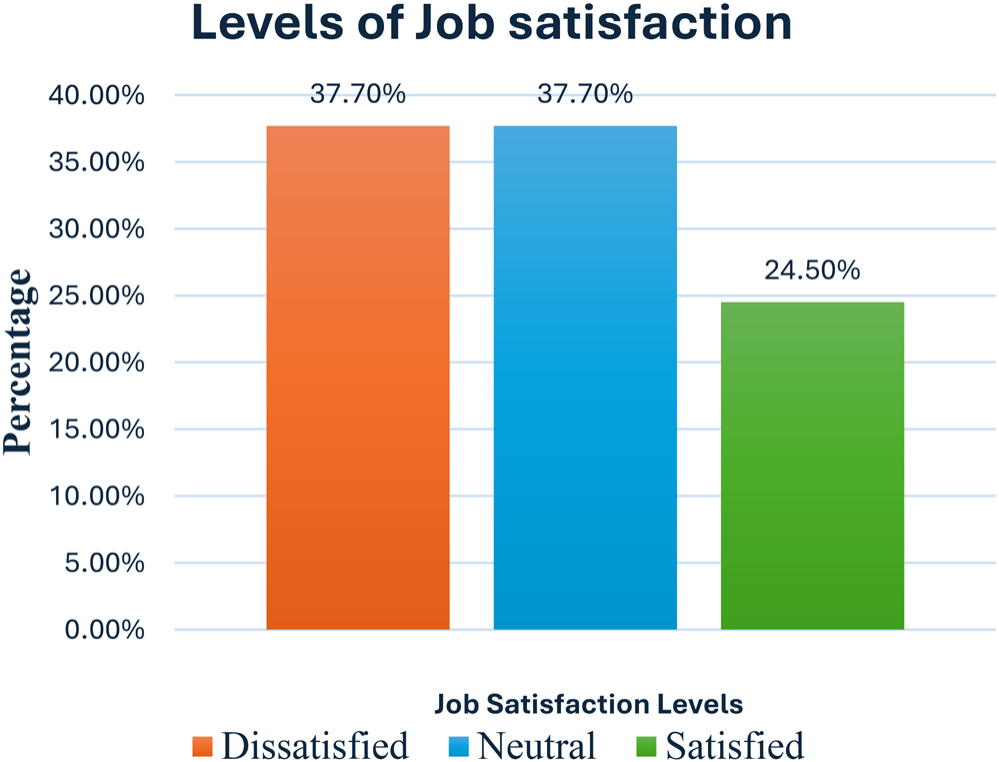


### 3.4 Correlation Between Job Satisfaction and Organisational Culture

Table 4 shows the correlation between the five sub‐scales of organisational culture variables to each other, as well as with job satisfaction. Correlation in this study was essential to inspect for multi‐collinearity before conducting regression analysis to determine the organisational culture determinants of job satisfaction. To cope with the issue of multi‐collinearity among independent variables, Hair et al. (2010) explained that the problem of multi‐collinearity occurs in a study if Pearson's *R*‐value is more than 0.90 (Hair et al. 2010). In our study, the highest Pearson's correlation value was between overall job satisfaction and nurse foundation for quality of Care, that is, 0.659. This was below 0.9, confirming that there was no problem of multi‐collinearity among the constructs. The results also showed that all the organisational culture variables were significant and positively correlated with nurses' job satisfaction.

**TABLE 4:** Pearson correlation.

Correlations						
	1	2	3	4	5	6
1. Overall, job satisfaction	1					
2. Nurse participation in hospital affairs (PHS)	0.579**	1				
3. Nurse foundation for quality of care (FQC)	0.440**	0.659**	1			
4. Nurse manager ability, leadership and support for nurses (LS)	0.482**	0.646**	0.638**	1		
5. Staffing and resource adequacy (SRA)	0.525**	0.446**	0.552**	0.473**	1	
6. Collegial nurse‐physician relationships (NPR)	0.453**	0.497**	0.558**	0.562**	0.534**	1

** Correlation is significant at the 0.01 level (2‐tailed), *n* = 212.

### 3.5 Regression Analysis: Organisational Culture Predictors of Job Satisfaction Among Nurses in Kenya

Structural equation modelling (SEM) analysis was conducted to identify the organisational culture predictors of job satisfaction among nurses in Kenya. The results, summarised in Table 5 include the hypothesised path, standardised regression weights (Beta Coefficients), standard errors, *T*‐values, confidence intervals, *p*‐values, and the hypothesis conclusion. The model confirmed two significant paths: Participation in hospital affairs (PHA), with a beta coefficient (β) of 0.409 (SE = 0.084, *T* = 5.396, *p* = < 0.001), and Staffing and Resource Adequacy (SRA), with a beta (β) of 0.311 (SE = 0.060, *T* = 4.665, *p* = < 0.001). These findings indicate that greater nurse participation in hospital affairs and adequate resources are associated with greater job satisfaction among nurses in Kenya. However, the paths for Foundation for quality of care (FQC) (β = −0.109, SE = 0.091, *T* = −1.370, *p* = 0.172), Leadership Support (LS) (β = 0.086, SE = 0.077, *T* = 1.119, *p* = 0.265), and Nurse‐Collegial‐Physician Relationships (NPR) (β = 0.096, SE = 0.064, *T* = 1.383, *p* = 0.168) were found to be insignificant. The overall model results (*R* ^2^ = 0.437, *F* = 31.983, df1 = 5, df2 = 206) explained 43.7% of the variation in job satisfaction among nurses, indicating a substantial impact of the identified organisational culture factors.

**TABLE 5:** SEM hypothesis testing‐regression results.

HYPOTHESIS	Hypothesised path	Beta coefficient	SE	*T*	95.0% Confidence interval for B		*p*	Hypothesis supported?
					Lower bound	Upper bound		
H1	JS ← PHA	0.409	0.084	5.396	0.287	0.618	0.000	YES
H2	JS ← FQC	−0.109	0.091	−1.370	−0.303	0.054	0.172	NO
H3	JS ← LS	0.086	0.077	1.119	−0.066	0.239	0.265	NO
H4	JS ← SRA	0.311	0.060	4.665	0.163	0.401	0.000	YES
H5	JS ← NPR	0.096	0.064	1.383	−0.038	0.216	0.168	NO
*R* ^2^ = 0.437								

*Note:* Dependent Variable: Nurses' Job Satisfaction. *R* ^2^ = 0.437, *F* = 31.983, df1 = 5, df2 = 206. Abbreviations: FQC, foundations for quality of care; JS, Job satisfaction; LS, leadership and support; NPR, nurses‐physician relations; PHA, participation in hospital affairs; SRA, staffing and resources adequacy.

## 4 Discussion

Nurses represent a fundamental Human Resource for Health (HRH) in any country of the world today, providing the essential healthcare services that are critical for the well‐being of the nation's populations. In developing countries like Kenya, this role is even more pronounced. Achievement of universal health coverage, the sustainable development goal 3, and Kenya's Vision 2030—central to the country's goals in healthcare delivery—hinge on adequate, stable, motivated, and more importantly, satisfied nursing personnel. Therefore, our present study was three‐fold: to find out how Kenyan nurses rate their organisational culture, their level of job satisfaction, and the organisational culture predictors of job satisfaction.

This study provides valuable and pertinent information illuminating the general picture of nursing culture in Kenya, a Sub‐Sahara African country in East Africa. Overall, organisational culture was rated slightly positive but extremely weak (*M* = 2.51). The five OC sub‐scales received mixed ratings, encompassing both negative and positive. Rated positive were *nursing foundations for quality‐of‐care scoring* (*M* = 2.64, SD = 1.01) *nurse manager ability, leadership, and support of nurses* (*M* = 2.63, SD = 1.00) and *collegial nurse‐physician relations* (*M* = 2.67, SD = 0.92). This positive scoring highlights key areas of strength in Kenya's healthcare system. Kenyan nurses are cognizant of the demand for quality healthcare service by patients and their relatives, and they consistently strive to meet these expectations. It was evident from the results, on their daily duty procedures, nurses in Kenya use nursing diagnosis, nursing care plans and some of their hospitals have quality assurance, and preceptor programmes for nurses. Nursing leadership appears supportive, engaging frontline nurses and recognising their work. This recognition may enhance job performance. There is also a strong nurses‐to‐nurses, nurse‐to‐physician and general inter‐cadre collaboration, teamwork, unity and general good working relationship as evidenced by the results on individual item ratings above mid‐point 2.5 in sub‐scale 5. This is a surprisingly powerful result worth noting from this study. The results were similar to those found in other countries like in Finland (Pursio et al. 2024), Spain (Fuentelsaz‐Gallego et al. 2013), Portugal (Leone et al. 2015) and Hong Kong (Choi et al. 2013) which rated OC and work environment slightly positive. However, differences were noted with studies from China (Wan et al. 2018), Saudi Arabia (Alsufyani et al. 2021), Brazil (Gasparino and Guirardello 2017) and result from over 13 countries from a systematic (Warshawsky and Havens 2011), where some studies had higher ratings of work environment culture, an indication of better healthcare systems in these nations than Kenya. In this study, the term ‘positive organisational culture’ is used to refer to a healthcare system characterised by strong leadership and organisational support, active nurse involvement, availability of career opportunities and professional development. It also includes effective communication, consultations, teamwork and collaboration among health teams, unity, commitment to high‐quality patient care and sufficient staff and resources. Conversely, ‘negative organisational culture’ signifies the absence of these organisational values. This study's result highlights areas of strengths and weaknesses within Kenya's healthcare system. Contrary to the three positively rated sub‐scales, two other sub‐scales *nurse participation in hospital affairs* (*M* = 2.45, SD = 1.03) and *staff and resource adequacy* (*M* = 2.11, SD 1.06) were rated negatively (poorly). This rating exposes major weaknesses in Kenya's healthcare system regarding nursing, leadership and opportunities. Findings show that nurses are not adequately involved in policy and governance as evidenced by scores of individual items in sub‐scale 1. The healthcare system in Kenya in the past had not given nurses equal opportunities compared to other medical professionals for example, doctors which seems to exist even in the current research (Kiiru, n.d.; Ndirangu et al. 2021). The management of the 6‐tier levels of healthcare facilities in Kenya for example, log out nurses from top management opportunities in many of these levels and have routinely been occupied by other healthcare professionals. Kenya lacks a proper legal nursing organogram from the lowest nursing management level to the highest (Ministry of Health Kenya 2022). From this study, it was evident that there were limited opportunities for professional development through promotions and career progression through academic opportunities. Kenya and other Sub‐Saharan Africa countries should therefore learn from developed countries who have nurse‐specific scholarships for career development for its nursing professions and implement such programmes. The study highlights the need for enhanced programmes, continuing education, and clear career pathways through regular and predictable promotions. The sub‐scale for *staff and resource adequacy* was rated the poorest. This is interpreted to mean there is an acute lack of staff, including nurses, to get work done in Kenyan hospitals as well as other important resources for nurses to function as further evidenced by the below regression analysis.

For the second objective, the findings established that only 24.5% of Kenyan nurses were satisfied with their jobs while 37.7% were unsatisfied. Another 37.7% remained neither satisfied nor dissatisfied.

### 4.1 Organisational Culture Predictors of Job Satisfaction Among Nurses in Kenya

This study's main objective was to determine organisational culture predictors of job satisfaction among nurses in Kenya. The results from Structural Equation Modelling (SEM) suggest that the five sub‐scales of organisational culture are crucial for nurses' job satisfaction. This is confirmed by the overall model, which indicates that these five variables account for 43.7% of job satisfaction variation. However, only nurses' participation in hospital affairs and staffing and resource adequacy were statistically significant predictors of job satisfaction. Increasing nurse involvement and participation in hospital affairs by one unit increases nurses' job satisfaction by 40.9% while a one‐unit increase in staff and resource adequacy raises nurses' job satisfaction by 30.1%. Nurses' participation in hospital affairs is the most critical value in the workplace as per this study. When nurses are involved in hospital affairs, they feel that their voice is heard and respected, management cares about them, and they are valued, emphasising their role as important team players. It brings a sense of empowerment (Choi et al. 2014) and allows nurses to contribute to policies that directly affect them. Furthermore, it enhances good communication, improves the work environment (Blake et al. 2013), and collaboration and reduces organisational silence and cynicism (Çaylak and Altuntaş 2017; Munir et al. 2018).

Another key predictor of nurses' job satisfaction found in this research was the adequacy of staff and resources. Kenya is among Sub‐Saharan countries with acute shortages (Donald Kokonya et al. 2014; Kimani and Gatimu 2023; Miseda et al. 2017; Sang et al. 2021). This shortage is both a real shortage (genuine inadequate qualified nursing professionals) and a pseudo‐shortage (qualified nurses available but not employed). Adequate staff ensure that nurses are not overburdened with excessive workloads, leading to a better work‐life balance and more flexible work schedules. This, in turn, reduces burnout, raises job morale, increases team cohesion, improves workplace safety (Rafferty et al. 2007), and enhances the quality of patient care (Van Bogaert et al. 2013). All these factors contribute to job satisfaction (Alharbi et al. 2020; Aziri 2011; Goh and Lopez 2016; Shah et al. 2018; Sharma 2017; Zhang et al. 2014). Likewise, resource adequacy encompassing medical and non‐medical supplies, equipment, support staff, Personal Protective Equipment (PPE) such as masks, gloves, and face shields, proper facility infrastructure including wards, beds, linen, operating rooms, care areas, well‐equipped break and restrooms, communication gadgets like hospital phones, educational materials, and transportation equipment like ambulances, hospital bus, stretchers and wheelchairs are very critical resources for nurses to function well. Despite the important role played by the adequacy of these resources, hospitals in developing countries like Kenya are ill‐equipped. *Kenya Health Facility Census Report* of September 2023 (Ministry of Health Kenya 2023) found that out of 11,147 examined hospitals, 77% had required infrastructure to offer outpatient services while 15% had proper required equipment. This situation is never better for in‐patient care and therefore action must be taken to enhance nurses' job satisfaction and quality service delivery.

Although Kenyan nurses rated *nursing foundations for quality‐of‐care scoring*, *nurse manager ability, leadership, and support of nurses* and *collegial nurse‐physician relations positively*, these factors were not statistically significantly associated with job satisfaction in this study. This may be due to several factors: their consistently high ratings have led to limited variability (a ceiling effect), reducing their statistical power; their influence might be indirect or mediated by other variables or may have become normalised aspects of the nurses' work environment, thus less impactful on job satisfaction. Additionally, cultural and systemic factors unique to the Kenyan healthcare contexts may shape how these elements are perceived by the nurses in relation to job satisfaction. These results suggest the need for broader investigation into other drivers of job satisfaction among nurses in this setting.

### 4.2 Study's Implication for Policy and Practice

Organisational culture and nurse job satisfaction are critical levers for transforming Kenya's healthcare system. This study reveals systemic weaknesses—especially poor nurse involvement, inadequate staffing, and lack of resources—that undermine morale for service delivery. Nurse managers, hospital leaders, policymakers, the Kenyan government and all other stakeholders should urgently prioritise the following policy interventions:

*Institutionalise and strengthen nurses' involvement in hospital affairs*: Establish a clear legal and management structure (organogram) that ensures that nurses are fully involved in hospital leadership, decision‐making, and all other roles in the day‐to‐day running of the hospital.

*Improve staffing levels*: Address both real and pseudo nurse shortages through targeted recruitment, deployment, and retention strategies.

*Enhancing resource adequacy*: Equip hospitals with enough medical supplies, personal protective equipment, proper infrastructure, and support staff to enable effective nursing care.

*Support career and professional development for nurses*: Introduce scholarships, predictable regular promotions, and continuing education programmes tailored for nurses.

*Foster supportive leadership*: Train nurse managers to build a positive organisational culture at work through recognition, communication and collaboration.

## 5 Conclusion

We conclude that organisational culture in Kenya is weak‐positive, while nurses' job satisfaction remains poor. From the five PES‐NWI sub‐scales of organisational culture examined in this study, only participation in hospital affairs and adequacy of staff and resources emerged as significant predictors of nurses' job satisfaction. The other factors—nursing foundation for quality of care, nurse manager ability, leadership and support of nurses, and collegial nurse‐physician relationship—were found to be insignificant. The present study is important to Kenya's national and county governments, non‐governmental organisations, faith‐based organisations, hospital management, and nurse managers among other health stakeholders. It underscores the need for Kenya to prioritise nurses' job satisfaction by creating a positive organisational culture for the country to achieve its goals of universal health coverage, Sustainable Development Goals, and Vision 2030; all its healthcare workers must be fully onboarded and satisfied.

### 5.1 Limitations of the Study

This study was limited to only organisational variables within the five sub‐scales and items of the PES‐NWI tool. Additionally, the three classifications of job satisfaction (satisfied, neutral and unsatisfied) may have contributed to the low levels of job satisfaction observed in the study. This study also used convenience sampling in selecting the respondents, which may introduce selection bias and generalisation of the study findings. Furthermore, data collection was conducted online, which may have excluded nurses with limited internet access or low digital literacy. Future research could broaden the scope of organisational culture values to include factors such as organisational justice, corruption, workplace laws and policies, discrimination, and remuneration. Job satisfaction can also be re‐classified into only ‘satisfied’ and ‘unsatisfied’ to reflect its true picture. To eradicate selection bias, future studies should use systematic sampling or other sampling methods that will eradicate bias for better generalisation.

## Author Contributions

**Evans Kasmai Kiptulon:** conceived the research idea, and participated in data collection, analysis and writing of the manuscript; **Anna Szőllősi:** participated in data collection and writing of the manuscript; **Zrínyi Miklós** and **Adrienn Ujváriné Siket:** acted as supervisor and advisor, reviewed, and edited this manuscript and approved it for publication.

## Ethics Statement

Ethical approval for this study was obtained from Moi University College of Health Sciences and the Moi Teaching and Referral Hospital Institutional Research and Ethics Committee, with approval number FAN:0004809. Additionally, written permission was granted by the Ministry of Health (MoH) Headquarters and Kenya National Commission for Science, Technology and Innovation (NACOSTI). Consent was obtained from each hospital. Ethical principles of respect for persons, beneficence, justice, confidentiality, non‐maleficence, privacy, and anonymity were adhered to. Participation was voluntary, with no consequences for participants who chose not to participate or decided to withdraw at any point during the study.

## Conflicts of Interest

The authors declare no conflicts of interest.

## Data Availability

In addition to the data analysed in this research, other datasets supporting the conclusions of this article are not publicly available but can be obtained from the corresponding author upon reasonable request.

## References

[nop270298-bib-0001] AbuAlRub, R. F. , and M. A. Nasrallah . 2017. “Leadership Behaviours, Organizational Culture and Intention to Stay Amongst Jordanian Nurses.” International Nursing Review 64, no. 4: 520–527. 10.1111/inr.12368. 28294309

[nop270298-bib-0002] Afulani, P. A. , L. Ongeri , J. Kinyua , M. Temmerman , W. B. Mendes , and S. J. Weiss . 2021. “Psychological and Physiological Stress and Burnout Among Maternity Providers in a Rural County in Kenya: Individual and Situational Predictors.” BMC Public Health 21, no. 1: 453. 10.1186/s12889-021-10453-0. 33676479 PMC7936594

[nop270298-bib-0003] Aghaei, H. A. , and Z. S. Asadi . 2020. “The Influence of Organizational Culture on Resilience by Mediatory Effects of Occupational Stress, Job Satisfaction, and Burnout in Nurses: Structural Equation ModelingModeling.” Iranian Red Crescent Medical Journal 22, no. 6: e102332. 10.5812/ircmj.102332.

[nop270298-bib-0004] Akinwale, O. E. , and O. J. George . 2020. “Work Environment and Job Satisfaction Among Nurses in Government Tertiary Hospitals in Nigeria.” Rajagiri Management Journal 14, no. 1: 71–92. 10.1108/RAMJ-01-2020-0002.

[nop270298-bib-0005] Alenezi, A. M. , A. Aboshaiqah , and O. Baker . 2018. “Work‐Related Stress Among Nursing Staff Working in Government Hospitals and Primary Health Care Centres.” International Journal of Nursing Practice 24, no. 5: e12676. 10.1111/ijn.12676. 30003631

[nop270298-bib-0006] Alharbi, A. A. , V. S. Dahinten , and M. MacPhee . 2020. “The Relationships Between Nurses' Work Environments and Emotional Exhaustion, Job Satisfaction, and Intent to Leave Among Nurses in Saudi Arabia.” Journal of Advanced Nursing 76, no. 11: 3026–3038. 10.1111/jan.14512. 32924146

[nop270298-bib-0007] Aloisio, L. D. , M. Coughlin , and J. E. Squires . 2021. “Individual and Organizational Factors of Nurses' Job Satisfaction in Long‐Term Care: A Systematic Review.” International Journal of Nursing Studies 123: 104073. 10.1016/j.ijnurstu.2021.104073. 34536909

[nop270298-bib-0008] Alsufyani, A. M. , K. E. Almalki , Y. M. Alsufyani , et al. 2021. “Impact of Work Environment Perceptions and Communication Satisfaction on the Intention to Quit: An Empirical Analysis of Nurses in Saudi Arabia.” PeerJ 9: e10949. 10.7717/peerj.10949. 33777522 PMC7980699

[nop270298-bib-0009] Al‐Surimi, K. , A. Almuhayshir , K. Y. Ghailan , and N. A. Shaheen . 2022. “Impact of Patient Safety Culture on Job Satisfaction and Intention to Leave Among Healthcare Workers: Evidence From Middle East Context.” Risk Management and Healthcare Policy 15: 2435–2451. 10.2147/RMHP.S390021. 36620517 PMC9811957

[nop270298-bib-0010] Arslan Yurumezoglu, H. , and G. Kocaman . 2016. “Predictors of Nurses' Intentions to Leave the Organisation and the Profession in Turkey.” Journal of Nursing Management 24, no. 2: 235–243. 10.1111/jonm.12305. 25900394

[nop270298-bib-0011] Aruasa, W. K. , L. K. Chirchir , and S. K. Chebon . 2019. “Determinants of Physicians and Nurses' Professional Satisfaction at the Moi Teaching and Referral Hospital, Eldoret, Kenya.” Journal of Health, Medicine and Nursing 64. https://core.ac.uk/download/pdf/234692818.pdf.

[nop270298-bib-0012] Ayalew, F. , S. Kibwana , S. Shawula , et al. 2019. “Understanding Job Satisfaction and Motivation Among Nurses in Public Health Facilities of Ethiopia: A Cross‐Sectional Study.” BMC Nursing 18, no. 1: 46. 10.1186/s12912-019-0373-8. 31636508 PMC6794848

[nop270298-bib-0013] Aziri, B. 2011. “Job Satisfaction: A Literature Review.” Management Research and Practice 3, no. 4: 77–86.

[nop270298-bib-0014] Banaszak‐Holl, J. , N. G. Castle , M. K. Lin , N. Shrivastwa , and G. Spreitzer . 2015. “The Role of Organizational Culture in Retaining Nursing Workforce.” Gerontologist 55, no. 3: 462–471. 10.1093/geront/gnt129. 24218146 PMC4542704

[nop270298-bib-0015] Bellou, V. 2010. “Organizational Culture as a Predictor of Job Satisfaction: The Role of Gender and Age.” Career Development International 15, no. 1: 4–19. 10.1108/13620431011020862.

[nop270298-bib-0016] Blake, N. , L. S. Leach , W. Robbins , N. Pike , and J. Needleman . 2013. “Healthy Work Environments and Staff Nurse Retention: The Relationship Between Communication, Collaboration, and Leadership in the Pediatric Intensive Care Unit.” Nursing Administration Quarterly 37, no. 4: 356–370. 10.1097/NAQ.0b013e3182a2fa47. 24022290

[nop270298-bib-0017] Boumans, N. P. , and J. A. Landeweerd . 1994. “Working in an Intensive or Non‐Intensive Care Unit: Does It Make Any Difference?” Heart and Lung: The Journal of Critical Care 23, no. 1: 71–79. 8150648

[nop270298-bib-0018] Bragadóttir, H. , B. J. Kalisch , B. G. Flygenring , and G. B. Tryggvadóttir . 2023. “The Relationship of Nursing Teamwork and Job Satisfaction in Hospitals.” SAGE Open Nursing 9: 237796082311750. 10.1177/23779608231175027. PMC10192802 37214231

[nop270298-bib-0019] Buchan, J. 2023. “Recover to Rebuild Investing in the Nursing Workforce for Health System Effectiveness Howard Catton.” Chief Executive Officer, International Council of Nurses International Council of Nurses.

[nop270298-bib-0020] Buffington, A. , J. Zwink , R. Fink , D. Devine , and C. Sanders . 2012. “Factors Affecting Nurse Retention at an Academic Magnet® Hospital.” Journal of Nursing Administration 42, no. 5: 273–281. 10.1097/NNA.0b013e3182433812. 22525291

[nop270298-bib-0022] Çaylak, E. , and S. Altuntaş . 2017. “Organizational Silence Among Nurses: The Impact on Organizational Cynicism and Intention to Leave Work.” Journal of Nursing Research 25, no. 2: 90–98. 10.1097/JNR.0000000000000139. 28277389

[nop270298-bib-0023] Chepkemoi, M. 2020. Nurses' Jon Satisfaction in Selected Public Hospitals in Kericho County, Kenya. Kenyatta University.

[nop270298-bib-0024] Choi, P. P. , W. M. Lee , S. S. Wong , and M. H. Tiu . 2022. “Competencies of Nurse Managers as Predictors of Staff Nurses' Job Satisfaction and Turnover Intention.” International Journal of Environmental Research and Public Health 19, no. 18: 11461. 10.3390/ijerph191811461. 36141733 PMC9517267

[nop270298-bib-0025] Choi, S. , I. Jang , S. Park , and H. Lee . 2014. “Effects of Organizational Culture, Self‐Leadership and Empowerment on Job Satisfaction and Turnover Intention in General Hospital Nurses.” Journal of Korean Academy of Nursing Administration 20, no. 2: 206. 10.11111/jkana.2014.20.2.206.

[nop270298-bib-0026] Choi, S. P. P. , K. Cheung , and S. M. C. Pang . 2013. “Attributes of Nursing Work Environment as Predictors of Registered Nurses' Job Satisfaction and Intention to Leave.” Journal of Nursing Management 21, no. 3: 429–439. 10.1111/j.1365-2834.2012.01415.x. 23409781

[nop270298-bib-0027] Chong, M. P. M. , Y. Shang , M. Richards , and X. Zhu . 2018. “Two Sides of the Same Coin? Leadership and Organizational Culture.” Leadership and Organization Development Journal 39, no. 8: 975–994. 10.1108/LODJ-05-2017-0122.

[nop270298-bib-0028] Dirdjo, M. M. , R. Syahab , E. Sureskiarti , and S. Suwanto . 2023. “Job Satisfaction and Nurse Performance.” Jurnal Ilmu Kesehatan 11, no. 1: 25–40. 10.30650/jik.v11i1.3700.

[nop270298-bib-0029] Ditlopo, P. , L. C. Rispel , P. Van Bogaert , and D. Blaauw . 2024. “The Impact of the Nurse Practice Environment, Workload, and Professional Support on Job Outcomes and Standards of Care at Primary Health Care Clinics in South Africa: A Structural Equation Model Approach.” International Journal of Nursing Studies Advances 7: 100241. 10.1016/j.ijnsa.2024.100241. 39351496 PMC11440313

[nop270298-bib-0030] Dolbier, C. L. , J. A. Webster , K. T. McCalister , M. W. Mallon , and M. A. Steinhardt . 2005. “Reliability and Validity of a Single‐Item Measure of Job Satisfaction.” American Journal of Health Promotion 19, no. 3: 194–198. 10.4278/0890-1171-19.3.194. 15693347

[nop270298-bib-0031] Donald Kokonya, K. A. , J. M. Mburu , D. M. Kathuku , et al. 2014. “Burnout Syndrome Among Medical Workers at Kenyatta National Hospital (KNH), Nairobi, Kenya.” 10.4172/2378-5756.1000142.

[nop270298-bib-0032] Eskola, N. S. , M. Roos , B. McCormack , P. Slater , N. Hahtela , and T. Suominen . 2016. “Workplace Culture Among Operating Room Nurses.” Journal of Nursing Management 24: 725–734. 10.1111/jonm.12376. 27113119

[nop270298-bib-0033] Fakunmoju, S. 2021. “Validity of Single‐Item Versus Multiple‐Item Job Satisfaction Measures in Predicting Life: Satisfaction and Turnover Intention.” Asia‐Pacific Journal of Management Research and Innovation 16, no. 3: 210–228. 10.1177/2319510X21997724.

[nop270298-bib-0034] Fuentelsaz‐Gallego, C. , M. T. Moreno‐Casbas , and E. González‐María . 2013. “Validation of the Spanish Version of the Questionnaire Practice Environment Scale of the Nursing Work Index.” International Journal of Nursing Studies 50, no. 2: 274–280. 10.1016/j.ijnurstu.2012.08.001. 22944284

[nop270298-bib-0035] Galletta, M. , I. Portoghese , M. P. Penna , A. Battistelli , and L. Saiani . 2011. “Turnover Intention Among Italian Nurses: The Moderating Roles of Supervisor Support and Organizational Support.” Nursing and Health Sciences 13, no. 2: 184–191. 10.1111/j.1442-2018.2011.00596.x. 21595810

[nop270298-bib-0036] Gary, F. , J. G. Voss , A. Y. Zhang , et al. 2023. “A Utilizing the Social Determinants of Health Model to Explore Factors Affecting Nurses' Job Satisfaction in Saudi Arabian Hospitals: A Systematic Review.” Healthcare 11: 2394. 10.3390/healthcare11172394. 37685428 PMC10487519

[nop270298-bib-0037] Gasparino, R. C. , and E. d. B. Guirardello . 2017. “Validation of the Practice Environment Scale to the Brazilian Culture.” Journal of Nursing Management 25, no. 5: 375–383. 10.1111/jonm.12475. 28303619

[nop270298-bib-0038] Gathongo, J. K. , and L. Ndimurwimo . 2020. “Strikes in Essential Services in Kenya: The Doctors, Nurses and Clinical Officers’.” Potchefstroom Electronic Law Journal 23: 1–25. 10.17159/1727-3781/2020/v23i0a5709.

[nop270298-bib-0039] Goffee, R. , and G. Jones . 1998. “The Character of a Corporation: How Your Company's Culture Can Make or Break Your Business.” 237.

[nop270298-bib-0040] Goh, Y. S. , and V. Lopez . 2016. “Job Satisfaction, Work Environment and Intention to Leave Among Migrant Nurses Working in a Publicly Funded Tertiary Hospital.” Journal of Nursing Management 24, no. 7: 893–901. 10.1111/jonm.12395. 27169747

[nop270298-bib-0041] Hair, J. F. , W. C. Black , and B. J. Babin . 2010. Multivariate Data Analysis (7th Edition). Pearson Education.

[nop270298-bib-0042] Hakami, A. , H. Almutairi , R. Alsulyis , T. Al Rrwis , and A. Al Battal . 2020. “The Relationship Between Nurses Job Satisfaction and Organizational Commitment.” Health Science Journal 14, no. 1: 1–5. 10.36648/1791-809X.14.1.692.

[nop270298-bib-0043] Haller, K. , W. Berends , and P. Skillin . 2018. “Organizational Culture and Nursing Practice: The Magnet Recognition Program® as A Framework for Positive Change.” Revista Médica Clínica Las Condes 29, no. 3: 328–335. 10.1016/J.RMCLC.2018.03.005.

[nop270298-bib-0044] Hayes, N. , C. Douglas , and A. Bonner . 2009. “Work Environment, Job Satisfaction, Stress and Burnout Among Haemodialysis Nurses.” Journal of Nursing Management 23, no. 5: 588–598. 10.1111/jonm.12184. 24372699

[nop270298-bib-0045] Hoppock, R. , and S. Spiegler . 1935. Job Satisfaction. Harper.

[nop270298-bib-0046] Hulkova, V. , M. Kilikova , and S. Sabo . 2024. “Organizational Culture of Health Care Facility as a Predictor of the Job Satisfaction of Nurses.” Clinical Social Work and Health Intervention 15, no. 2: 32–36. 10.22359/cswhi_15_2_06.

[nop270298-bib-0047] Hwang, E. 2019. “Effects of the Organizational Culture Type, Job Satisfaction, and Job Stress on Nurses' Happiness: A Cross‐Sectional Study of the Long‐Term Care Hospitals of South Korea.” Japan Journal of Nursing Science 16, no. 3: 263–273. 10.1111/jjns.12235. 30259668

[nop270298-bib-0048] Judge, T. A. 2017. “Promote Job Satisfaction Through Mental Challenge.” In The Blackwell Handbook of Principles of Organizational Behaviour, 77–92. Wiley. 10.1002/9781405164047.ch6.

[nop270298-bib-0049] Kaguthi, G. K. , V. Nduba , and M. B. Adam . 2020. “The Impact of the Nurses', Doctors' and Clinical Officer Strikes on Mortality in Four Health Facilities in Kenya.” BMC Health Services Research 20, no. 1: 469. 10.1186/s12913-020-05337-9. 32456634 PMC7249343

[nop270298-bib-0050] Kaushik, A. , S. Ravikiran , K. Suprasanna , M. Nayak , K. Baliga , and S. Acharya . 2021. “Depression, Anxiety, Stress and Workplace Stressors Among Nurses in Tertiary Health Care Settings.” Indian Journal of Occupational And Environmental Medicine 25, no. 1: 27–32. 10.4103/IJOEM.IJOEM_123_20. 34295059 PMC8259589

[nop270298-bib-0051] Kiiru, S. N. n.d. “The Influence of Organizational Culture on Perfomance of Hospitals in Nairobi, Kenya.”

[nop270298-bib-0052] Kimani, R. W. , and S. M. Gatimu . 2023. “Nursing and Midwifery Education, Regulation and Workforce in Kenya: A Scoping Review.” International Nursing Review 70: 444–455. 10.1111/inr.12840. 36970943

[nop270298-bib-0053] Kirigia, J. M. , A. R. Gbary , L. K. Muthuri , J. Nyoni , and A. Seddoh . 2006. “The Cost of Health Professionals' Brain Drain in Kenya.” BMC Health Services Research 6: 89. 10.1186/1472-6963-6-89. 16846492 PMC1538589

[nop270298-bib-0054] Koberg, C. S. , and L. H. Chusmir . 1987. “Organizational Culture Relationships With Creativity and Other Job‐Related Variables.” Journal of Business Research 15, no. 5: 397–409. 10.1016/0148-2963(87)90009-9.

[nop270298-bib-0055] Körner, M. , M. A. Wirtz , J. Bengel , and A. S. Göritz . 2015. “Relationship of Organizational Culture, Teamwork and Job Satisfaction in Interprofessional Teams Organization, Structure and Delivery of Healthcare.” BMC Health Services Research 15, no. 1: 243. 10.1186/s12913-015-0888-y. 26099228 PMC4477418

[nop270298-bib-0056] Labrague, L. J. , D. M. McEnroe‐Petitte , D. Gloe , K. Tsaras , D. L. Arteche , and F. Maldia . 2017. “Organizational Politics, Nurses' Stress, Burnout Levels, Turnover Intention and Job Satisfaction.” International Nursing Review 64, no. 1: 109–116. 10.1111/INR.12347. 27995623

[nop270298-bib-0057] Lake, E. T. 2002. “Development of the Practice Environment Scale of the Nursing Work Index.” Research in Nursing and Health 25, no. 3: 176–188. 10.1002/NUR.10032. 12015780

[nop270298-bib-0058] Leone, C. , L. Bruyneel , J. E. Anderson , et al. 2015. “Work Environment Issues and Intention‐To‐Leave in Portuguese Nurses: A Cross‐Sectional Study.” Health Policy 119, no. 12: 1584–1592. 10.1016/j.healthpol.2015.09.006. 26474746

[nop270298-bib-0059] Lin, B. Y.‐J. , T. T. H. Wan , C.‐P. C. Hsu , F.‐R. Hung , C.‐W. Juan , and C.‐C. Lin . 2012. “Relationships of Hospital‐Based Emergency Department Culture to Work Satisfaction and Intent to Leave of Emergency Physicians and Nurses.” Health Services Management Research 25, no. 2: 68–77. 10.1258/hsmr.2012.012011. 22673696

[nop270298-bib-0060] McDonald, K. , L. B. Rubarth , and L. J. Miers . 2012. “Job Satisfaction of Neonatal Intensive Care Nurses.” Advances in Neonatal Care 12, no. 4: E1–E8. 10.1097/ANC.0b013e3182624eb1. 22864007

[nop270298-bib-0061] Ministry of Health Kenya . 2022. “National Nursing and Midwifery Policy Kenya 2022–2032”.

[nop270298-bib-0062] Ministry of Health Kenya . 2023. “Kenya Health Facility Census Report”.

[nop270298-bib-0063] Miseda, M. H. , S. O. Were , C. A. Murianki , M. P. Mutuku , and S. N. Mutwiwa . 2017. “The Implication of the Shortage of Health Workforce Specialist on Universal Health Coverage in Kenya.” Human Resources for Health 15, no. 1: 80. 10.1186/s12960-017-0253-9. 29191247 PMC5710014

[nop270298-bib-0064] MOH KENYA . 2020. “Kenya's Health Sector‐Number of Hospitals”.

[nop270298-bib-0065] Munir, Y. , M. M. Ghafoor , and A. M. D. Rasli . 2018. “Perception of Ethical Climate and Turnover Intention Among Nursing Staff: Does Organizational Cynicism Mediate?” International Journal of Human Rights in Healthcare 11, no. 5: 319–332. 10.1108/IJHRH-07-2017-0028.

[nop270298-bib-0066] Nagel, C. , and K. Nilsson . 2022. “Nurses' Work‐Related Mental Health in 2017 and 2020—A Comparative Follow‐Up Study Before and During the COVID‐19 Pandemic.” International Journal of Environmental Research and Public Health 19, no. 23: 15569. 10.3390/IJERPH192315569. 36497643 PMC9738150

[nop270298-bib-0067] Nantsupawat, A. , W. Kunaviktikul , R. Nantsupawat , O. A. Wichaikhum , H. Thienthong , and L. Poghosyan . 2017. “Effects of Nurse Work Environment on Job Dissatisfaction, Burnout, Intention to Leave.” International Nursing Review 64, no. 1: 91–98. 10.1111/inr.12342. 27882573

[nop270298-bib-0068] Ndirangu, E. W. , A. M. Sarki , C. Mbekenga , and G. Edwards . 2021. “Professional Image of Nursing and Midwifery in East Africa: An Exploratory Analysis.” BMC Nursing 20, no. 1: 37. 10.1186/s12912-020-00531-w. 33676509 PMC7936462

[nop270298-bib-0069] Nyang'echi, E. N. , J. O. Osero , and A. Yitambe . 2019. “Job Satisfaction Among Nurses in Nyamira County, Kenya.” European Journal of Pharmaceutical and Medical Research 6, no. 7: 12–16.

[nop270298-bib-0093] Olusegun Emmanuel, A. , and O. J. George . 2020. “Work Environment and Job Satisfaction Among Nurses in Government Tertiary Hospitals in Nigeria.” Rajagiri Management Journal. 10.1108/RAMJ-01-2020-0002.

[nop270298-bib-0070] Oshagbemi, T. 1999. “Overall Job Satisfaction: How Good Are Single Versus Multiple‐Item Measures?” Journal of Managerial Psychology 14, no. 5: 388–403. 10.1108/02683949910277148.

[nop270298-bib-0071] Park, J. S. , and T. H. Kim . 2009. “Do Types of Organizational Culture Matter in Nurse Job Satisfaction and Turnover Intention?” Leadership in Health Services 22, no. 1: 20–38. 10.1108/17511870910928001.

[nop270298-bib-0072] Pursio, K. , P. Kankkunen , S. Mikkonen , and T. Kvist . 2024. “Organizational Characteristics of Nursing Practice Environments Related to Registered Nurses' Professional Autonomy and Job Satisfaction in Two Finnish Magnet‐Aspiring Hospitals: Structural Equation Modeling Study.” BMC Nursing 23, no. 1: 100. 10.1186/s12912-024-01772-9. 38321511 PMC10845793

[nop270298-bib-0073] Queen, M. , K. Van Der , and W. Annatjie . 2022. “The Current Workplace Culture in a South African Nursing Education Institution.” Gender and Behaviour 20, no. 2.

[nop270298-bib-0074] Rafferty, A. M. , S. P. Clarke , J. Coles , et al. 2007. “Outcomes of Variation in Hospital Nurse Staffing in English Hospitals: Cross‐Sectional Analysis of Survey Data and Discharge Records.” International Journal of Nursing Studies 44, no. 2: 175–182. 10.1016/j.ijnurstu.2006.08.003. 17064706 PMC2894580

[nop270298-bib-0075] Roza, N. , Y. Supriyati , and K. Kadir . 2022. “Organizational Culture, Career Development, Job Satisfaction and Nurse Performance at Batam City Hospital.” In Eighth Padang International Conference on Economics Education, Economics, Business and Management, Accounting and Entrepreneurship (PICEEBA‐8 2021), 362–367. Atlantis Press.

[nop270298-bib-0076] Sang, J. C. , O. F. Newa , S. Murei , and W. Aruwasa . 2021. “Exodus of Healthcare Professionals: Antecedents of Occupational Turnover Among Nurses in Kenya.” International Journal of Academic Research in Business and Social Sciences 11, no. 2: 695–716. 10.6007/ijarbss/v11-i2/8126.

[nop270298-bib-0077] Schein, E. H. 2018. Organizational Culture and Leadership. 3rd ed. Wiley.

[nop270298-bib-0078] Sexton, J. B. , R. L. Helmreich , T. B. Neilands , et al. 2006. “The Safety Attitudes Questionnaire: Psychometric Properties, Benchmarking Data, and Emerging Research.” BMC Health Services Research 6, no. 1: 44. 10.1186/1472-6963-6-44. 16584553 PMC1481614

[nop270298-bib-0079] Shah, S. M. M. , R. Ali , A. S. Dahri , N. A. Brohi , and Z. A. Maher . 2018. “Determinants of Job Satisfaction Among Nurses: Evidence From South Asian Perspective.” International Journal of Academic Research in Business and Social Sciences 8, no. 5: 19–26. 10.6007/IJARBSS/v8-i5/4082.

[nop270298-bib-0080] Sharma, P. 2017. “Organizational Culture as a Predictor of Job Satisfaction: The Role of Age and Gender.” Management: Journal of Contemporary Management Issues 22, no. 1: 35–48. 10.30924/mjcmi/2017.22.1.35.

[nop270298-bib-0082] Statista . 2022. “Number of Nurses in Kenya From 2016 to 2022, by Category”.

[nop270298-bib-0083] Tsai, Y. 2011. “Relationship Between Organizational Culture, Leadership Behavior and Job Satisfaction.” BMC Health Services Research 11: 98. 10.1186/1472-6963-11-98. 21569537 PMC3123547

[nop270298-bib-0084] Van Bogaert, P. , C. Kowalski , S. M. Weeks , D. Van heusden , and S. P. Clarke . 2013. “The Relationship Between Nurse Practice Environment, Nurse Work Characteristics, Burnout and Job Outcome and Quality of Nursing Care: A Cross‐Sectional Survey.” International Journal of Nursing Studies 50, no. 12: 1667–1677. 10.1016/j.ijnurstu.2013.05.010. 23777786

[nop270298-bib-0085] Wan, Q. , Z. Li , W. Zhou , and S. Shang . 2018. “Effects of Work Environment and Job Characteristics on the Turnover Intention of Experienced Nurses: The Mediating Role of Work Engagement.” Journal of Advanced Nursing 74, no. 6: 1332–1341. 10.1111/jan.13528. 29350781

[nop270298-bib-0086] Wang, L. , H. Tao , C. H. Ellenbecker , and X. Liu . 2012. “Job Satisfaction, Occupational Commitment and Intent to Stay Among Chinese Nurses: A Cross‐Sectional Questionnaire Survey.” Journal of Advanced Nursing 68, no. 3: 539–549. 10.1111/j.1365-2648.2011.05755.x. 21722170

[nop270298-bib-0087] Wanous, J. P. , A. E. Reichers , and M. J. Hudy . 1997. “Overall Job Satisfaction: How Good Are Single‐Item Measures?” Journal of Applied Psychology 82, no. 2: 247–252. 10.1037/0021-9010.82.2.247. 9109282

[nop270298-bib-0088] Warshawsky, N. E. , and D. S. Havens . 2011. “Global Use of the Practice Environment Scale of the Nursing Work Index.” Nursing Research 60, no. 1: 17–31. 10.1097/NNR.0b013e3181ffa79c. 21127450 PMC3021172

[nop270298-bib-0089] Widodo, D. S. , N. Hidayah , and S. D. Handayani . 2021. “Effect of Organizational Culture, Pay Satisfaction, Job Satisfaction on Nurse Intention to Leave at Private Hospital Type D in Bantul.” Journal: JMMR (Jurnal Medicoeticolegal dan Manajemen Rumah Sakit) 10, no. 2: 207–216. 10.18196/jmmr.v10i2.11408.

[nop270298-bib-0090] Wilson Van Voorhis, C. R. , and B. L. Morgan . 2007. “Understanding Power and Rules of Thumb for Determining Sample Sizes.” Tutorial in Quantitative Methods for Psychology 3, no. 2: 43–50. 10.20982/tqmp.03.2.p043.

[nop270298-bib-0091] Zahednezhad, H. , M. A. Hoseini , A. Ebadi , P. Farokhnezhad Afshar , and R. Ghanei Gheshlagh . 2021. “Investigating the Relationship Between Organizational Justice, Job Satisfaction, and Intention to Leave the Nursing Profession: A Cross‐Sectional Study.” Journal of Advanced Nursing 77, no. 4: 1741–1750. 10.1111/jan.14717. 33305518

[nop270298-bib-0092] Zhang, L. f. , L. m. You , K. Liu , et al. 2014. “The Association of Chinese Hospital Work Environment With Nurse Burnout, Job Satisfaction, and Intention to Leave.” Nursing Outlook 62, no. 2: 128–137. 10.1016/j.outlook.2013.10.010. 24345617 PMC3959248

